# Advancing to a Circular Economy: three essential ingredients for a comprehensive policy mix

**DOI:** 10.1007/s11625-017-0502-9

**Published:** 2017-11-07

**Authors:** Leonidas Milios

**Affiliations:** International Institute for Industrial Environmental Economics, Lund University, P. O. Box 196, Tegnersplatsen 4, 221 00 Lund, Sweden

**Keywords:** Circular Economy, Resource efficiency, Policy

## Abstract

Material resources exploitation and the pressure on natural ecosystems have raised concerns over potential future resource risks and supply failures worldwide. Interest in the concept of Circular Economy has surged in recent years among policy makers and business actors. An increasing amount of literature touches upon the conceptualisation of Circular Economy, the development of ‘circular solutions’ and circular business models, and policies for a Circular Economy. However, relevant studies on resource efficiency policies mostly utilise a case-by-case or sector-by-sector approach and do not consider the systemic interdependencies of the underlying operational policy framework. In this contribution, a mapping of the existing resource policy framework in the European Union (EU) is undertaken, and used as a basis for identifying policy areas that have been less prominent in influencing material resource efficiency. Employing a life cycle approach, policies affecting material efficiency in the production and consumption stages of a product have been found to be poorly utilised so far in the EU. Taking this as a point of departure, three policy areas that can contribute to closing material loops and increasing resource efficiency are thoroughly discussed and their application challenges are highlighted. The three policy areas are: (1) policies for reuse, repair and remanufacturing; (2) green public procurement and innovation procurement; and (3) policies for improving secondary materials markets. Finally, a potential policy mix, including policy instruments from the three mentioned policy areas—together with policy mixing principles—is presented to outline a possible pathway for transitioning to Circular Economy policy making.

## Introduction

In an increasingly expanding global economy within a resource-constrained world, concerns over the exploitation and potential future shortage of the earth’s natural resources grow rapidly worldwide. Resource extraction and use is further linked to emissions and waste generation, which contribute to adverse environmental pressures (Hashimoto et al. 2012). The global ecological footprint of human activities has increased from less than one planet Earth in 1961 to more than 1.4 planet Earths in 2005 (Galli et al. 2012) and is expected to grow further to two planet Earths around 2030 (Moore et al. 2012), while at the same time studies on planetary boundaries demonstrate that the ability of natural ecosystems to endure stress and regenerate is limited (Rockström et al. 2009).

Nevertheless, instead of maintaining the global level of material use close to a sustainable level—estimated to be around 8 tonnes of resource use per capita (Mont et al. 2013)—the material throughput in society is further aggravated by the steady decline of product life spans (Bakker et al. 2014). Linear economic activities (i.e. where resources are rapidly consumed and production processes do not account for their unsustainable exploitation neither their recovery) rely exclusively on the shrinking pool of earth’s natural resources and impose potential risks in the long run to the society as a whole. If linear production and consumption practices are complemented—and gradually substituted—by circular material flows, substantial resource efficiency improvements can be achieved (Ellen MacArthur Foundation 2012). Products and services, therefore, need to be designed purposefully with material resource efficiency in mind, assuming a life cycle perspective. Resource saving strategies for reuse, repair, remanufacturing and recycling of products and their components are required in this ‘new’ Circular Economy, which would enable products to gain a ‘new life’.

**Fig. 1:**
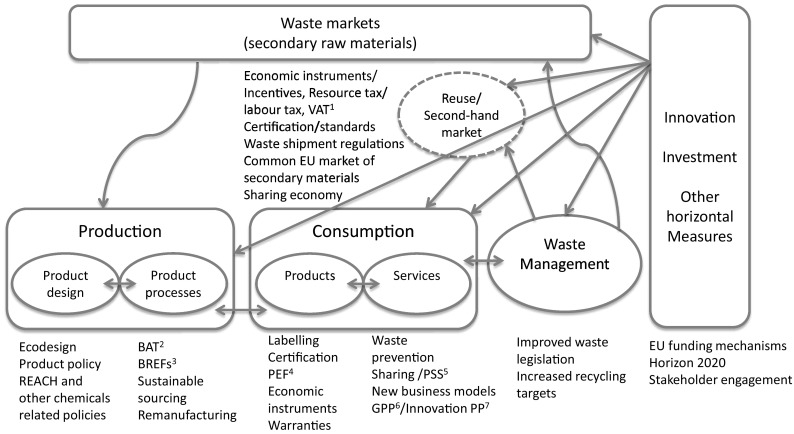
EU policy landscape. ^1^ Value Added Tax; ^2^ Best Available Techniques; ^3^ BAT Reference documents; ^4^ Product Environmental Footprint; ^5^ Product-Service System; ^6^ Green Public Procurement; ^7^ Public Procurement

Murray et al. (2017) suggest that the Circular Economy represents the most recent attempt to conceptualise the integration of economic activity with environmental and resource concerns in a sustainable way. In other words, the concept of Circular Economy is combining old and well-established notions of resource efficiency while making explicit the economic aspect of saving resources and the potential gains it accrues. Globally, the resource efficiency policy agenda is a relatively new area, which has seen a rapid development and popularity since the beginning of the twenty-first century, mainly due to changes experienced in global commodity markets (European Commission 2015b). In the last 15 years, massive global awareness and policy efforts, concerning the efficient use of resources, are observed for instance in Japan, European Union and China (Ghisellini et al. 2016). Recently, the European Commission finalised a comprehensive action plan for transitioning to a Circular Economy in Europe, describing an array of necessary policy interventions across the life cycle of products that should be considered in the short/medium term of policy development. Figure 1 outlines the plurality of measures in the new European Circular Economy Action Plan [COM (2015) 614 final], and illustrates the complexity of interactions of measures within a life cycle perspective.

Exemplified by the complexity outlined in Fig. 1, a novel approach in policy development is required; one that dictates a rather holistic policy view at systems level. Thorough policy making needs to understand the underlying premises of the problem and target its relevant aspects. Therefore, policy aiming to conserve resources and increase material resource efficiency in production and consumption is required to identify and intervene in all relevant life-cycle stages of products. Life-cycle stages that have received considerable attention so far, and as a result demonstrate some improvements, are the production stage and to some extent the waste management and product design stages (Mont and Bleischwitz 2007). Evidence from analyses by the European Environment Agency (EEA) demonstrates that environmental pressures of European consumption are steadily increasing, despite production-related technology gains. (EEA 2012, 2014).

Against this background, this paper seeks to identify policy areas currently underutilised at EU level—or policy gaps—and to discuss the potential of upscaling and integrating such policies into a resource-efficiency oriented and comprehensive policy framework within a Circular Economy paradigm. The analysis takes a life cycle approach in identifying policy deficiencies at different life cycle stages of a product, and continues by thoroughly examining these deficiencies through relevant literature review. Finally, literature around the principles of policy mixing is reviewed to inform the final position of this paper, having in mind the complexity of systems theory and life cycle thinking.

The particular focus of this contribution lies on the specific policy areas identified through the policy framework analysis in “Policy landscape in the European Union”. However, a brief review of the concept and application of CE so far will be presented in “Circular Economy: what does it really mean?”, based on the rapidly developing literature related to CE and its application in different geo-political jurisdictions (e.g. Ghisellini et al. 2016; Andersen 2007; Blomsma and Brennan 2017; Bocken et al. 2016; Mathews et al. 2011; McDowall et al. 2017; Murray et al. 2017; Skene 2017; Stahel 2016; Winans et al. 2017; Feng and Yan 2007; Zink and Geyer 2017), economic sectors (Lieder and Rashid 2016; Kristensen et al. 2016; Esa et al. 2017; Ness and Xing 2017; Pomponi and Moncaster 2017), and different sustainability dimensions (e.g. Andrews 2015; Geissdoerfer et al. 2017; Gregson et al. 2015; Moreau et al. 2017; Sauvé et al. 2016), aiming to provide the necessary background context.

The research methodology used for answering the objectives of this paper includes an extensive literature review of academic sources in related thematic areas and relevant policies, at EU and national level. The literature review commenced with searching for scientific publications using relevant keywords in databases such as Web of Science, Scopus, and Google Scholar. Then snowballing technique was used (in terms of keywords, authors’ names and journal titles) to expand the preliminary reference list for all the thematic areas and policies identified. Furthermore, official EU and national policy documents, as well as EU and national documentation for supporting policy decisions (such as preparatory studies, impact assessments and other related reports) were used for drawing a complete policy map of the current resource policy framework in the EU. For mapping the existing policy landscape in the EU, all regulations found at the EU law directory EUR-Lex^1^ were scrutinized and only those relevant to material resource efficiency were selected and respectively positioned within the life cycle stage they primarily regulate (see Table 1).

**Table 1:** Policies affecting resource efficiency in different life cycle stages of a product, at EU-28 level

Life cycle stage	Production	Use/consumption	Waste management
Mandatory	(Batteries and waste batteries Directive 2013/56/EU) (WEEE Directive 2012/19/EU) (RoHS Directive 2011/65/EU) Ecodesign Directive 2009/125/EC^a^ Packaging and waste packaging Directive 94/62/EC (Standardisation Regulation (EU) No 1025/2012) (Marketing of construction products Regulation (EU) No 305/2011) (REACH Regulation (EC) No 1907/2006^a^)	(Labelling of energy-related products Directive 2010/30/EU) Ecodesign Directive 2009/125/EC^a^ (Sale of consumer goods and associated guarantees Directive 1999/44/EC)	Waste Framework Directive 2008/98/EC Batteries and waste batteries Directive 2013/56/EU Plastic bags Directive (EU) 2015/720 WEEE Directive 2012/19/EU RoHS Directive 2011/65/EU Waste from extractive industries Directive 2006/21/EC ELV Directive 2000/53/EC Landfill Directive 1999/31/EC Packaging and waste packaging Directive 94/62/EC Shipments of waste Regulation (EU) No 660/2014 (REACH Regulation (EC) No 1907/2006^a^)
Voluntary	(Public procurement Directive 2014/24/EU) (Ecolabel Regulation (EC) No 66/2010)	(Public procurement Directive 2014/24/EU) (Ecolabel Regulation (EC) No 66/2010)	

Policies in parenthesis have only partial or indirect effect on CE

^a^The ecodesign directive and REACH regulation serve as a policy framework out of which specific implementing measures are formulated and applied by case (product group or chemical compound respectively). To date, the application of ecodesign focused primarily on energy efficiency measures and material resource efficiency appears very limited (for an overview of ecodesign processes in relation to material resource efficiency see Bundgaard et al. 2017)

The article begins by analysing fundamental elements of the Circular Economy in “Circular Economy: what does it really mean?” presenting a basic understanding of the term, its special characteristics and its limitations, serving as a conceptual background to the following policies review. The current policy landscape in the EU is analysed in “Policy landscape in the European Union”, and gaps are identified, which creates the basis for discussion about potential policies in “Three policy options for advancing to a Circular Economy”. The three major policy areas which correspond to the gaps that have been identified in “Policy landscape in the European Union” are thoroughly discussed in “Three policy options for advancing to a Circular Economy”. “Policy mix for an effective circular approach” outlines the need for combining the proposed policy areas and exemplifies a way to create effective policy packages. A practical application of policy mixing for increasing resource efficiency was outlined by Ekvall et al. (2016) in a case study of a ‘metals use’ policy mix in EU-27. However, “Policy mix for an effective circular approach” presents a theory based systemic approach in policy mixing that can be applied in a variety of cases and scenarios. Finally, “Conclusions” concludes by pointing out potential areas for future research concerning the development and uptake of policies and policy packages for CE.

## Circular Economy: what does it really mean?

### The concept and its limitations

The most widely used definition of the Circular Economy is the one formulated by the Ellen MacArthur Foundation in the early 2010s, ‘[…] an industrial system that is restorative or regenerative by intention and design. It replaces the end‐of‐life concept with restoration, shifts towards the use of renewable energy, eliminates the use of toxic chemicals, which impair reuse, and aims for the elimination of waste through the superior design of materials, products, systems, and, within this, business models’ (Ellen MacArthur Foundation 2012, p:7). While this definition implies the generic application of the concept, it might be a little problematic when it comes to informing policy processes, as it includes specialised terms that are rather challenging to conceptualise and operationalise at a policy level. Terms such as *‘*restorative’ and ‘regenerative’ are not clear enough in a policy context, while ‘superior design’ is rather an arbitrary term not related to any criteria or assessment. In the Circular Economy Action Plan of the European Commission (COM(2015) 614 final) there is no ‘official’ definition of the concept, but the understanding of the European Commission regarding the concept of Circular Economy can be deciphered in the first few lines of the CE Action Plan, ‘[…] circular economy, where the value of products, materials and resources is maintained in the economy for as long as possible, and the generation of waste minimised.’ This definition seems to appeal stronger among policy and business circles. Government agencies work already towards the objectives outlined in this definition, while the World Business Council for Sustainable Development recently released a strategy document presenting exactly the same EU definition as their understanding for the Circular Economy (WBCSD 2016).

Circular Economy encompasses and builds upon a number of complementary approaches, including ecodesign (Brezet and van Hemel 1997), lean manufacturing (Nakajima 2000), industrial ecology (Erkman 1997), industrial symbiosis (Ehrenfeld and Gertler 1997), cradle-to-cradle (Stahel and Reday-Mulvey 1981), life cycle thinking (Dalhammar 2015), waste-to-resources (Kama 2015), sustainable consumption (Mont and Heiskanen 2015), dematerialisation (Andrews 2015), functional economy (Stahel 1997), and product-service systems (Tukker and Tischner 2006).

Stahel (2013) argues that the concept has not yet reached any wide implementation stage, because policymakers and economic actors know neither the basic principles of Circular Economy, nor their impact on the economy. To overcome this general lack of knowledge, Stahel (2013) outlines a set of principles that would apply in a Circular Economy: (a) the smaller the resource circulation (activity-wise and geographically) the more profitable and resource efficient; (b) material loops are continuous, therefore, materials constantly circulate in the economy and feed into new production processes, minimising potential waste; (c) maintaining the value, quality and performance of goods; (d) the efficiency of managing stocks in CE increases with a decreasing flow speed; (e) extending ownership is a cost-efficient strategy, as reuse, repair and remanufacturing without ownership changes saves on transaction costs; and (f) CE requires the existence of well-functioning second hand product and secondary materials markets. Skene (2017) presents a similar set of principles, and complements further with (g) elimination of toxic substances and (h) renewable energy use.

Sustaining the virtuous loops of production and consumption in the economy by keeping materials in the economy for as long as possible might pose a particular problem, as inevitably material circulation reaches its limits, while the possibility of rebound effects seems imminent (Zink and Geyer 2017). At some point, the extra cost of improving and refining further a circular material flow will exceed the corresponding benefits to society. Specifically, a Circular Economy should promote loops when socially desirable and efficient for as long as the benefit is greater than or equal to the cost (Andersen 2007). For this reason, there is a need to address three underlying conditions which pose significant challenges towards achieving a Circular Economy:

1. Global population is increasing at fast pace, and therefore, there is no chance to fully close material circles without reducing material intensity in production and consumption patterns through efficiency and sufficiency strategies (Alcott 2008). This reflects the need for equal focus on production and consumption policies, triggering a wider behavioural shift in the modern society.
2. 100% recyclability is not possible (governed by physics laws) and endless reuse and recycling is also not possible because a range of materials lose their properties over time (with the exception of metals and some minerals). Therefore, materials are downcycled at some point in their subsequent circulations in the economy, and ultimately are discarded (Daly 1977; Faber et al. 1987). This signifies the imperative of product life extension efforts through appropriately designed policies, innovative business models and technological improvements.
3. Current material flows within the economy are not sufficient to fulfil the material demand resulting from points (1) and (2). Therefore, a need to capitalise on “historically” lost resources which might lie hidden in old landfills or stored out-of-use somewhere (e.g. old mobiles) will emerge (i.e. urban mining). Ultimately, highly efficient and effective recovery systems for all possible valuable materials in the society, and their reintroduction to the economy, are needed for fulfilling the vision of CE.

### Application and complexity in practice

First notions of Circular Economy elements in national strategic development can be traced to the 1980s and 1990s in German and Japanese policy, influenced by the intriguing and then ‘new’ concept of a closed-loop economy (Moriguchi 2007). These policies, in turn, inspired China to devise the Circular Economy as its major framework for industrial development, delivering increased economic growth with decreased environmental impacts (Yuan et al. 2006; Yong 2007; Feng and Yan 2007).

However, the application of CE in different geopolitical jurisdictions differs to some extent. The implementation of CE in China, Japan, and Europe although rooted in the basic principles of CE, it seems to have taken a slightly different approach. CE in China comes as a direct outcome of the national political strategy (top down approach), and its implementation is structured following both a horizontal and a vertical approach (Feng and Yan 2007). CE policies in China target the different levels of industrial/societal systems and seem to draw directly from theories of Industrial Symbiosis (IS) and Industrial Ecology (IE) systems. These include four industrial sectors (i.e. eco-industry, eco-farming, green services, and the reuse and recycling industry) and are applied in three scales of material cycles—small cycles at the enterprise level (micro-level), medium cycles at the industrial system level (meso-level), and a large cycle in society (macro-level) (Yong 2007).

On the other hand, Ghisellini et al. (2016) argue that the main focus in the EU was primarily put on policies promoting efficient and effective waste management, aiming at improving recycling rates in Europe, and consequently aiming at reaping the benefits of higher resource circulation in the economy. Although this latter part was not directly regulated by the policies in place, it was largely expected indirectly as a result from the policies implementation. Similarly, Japan appears to have adopted a rather inclusive approach, embracing the 3R principles (Reduce, Reuse, Recycle) and establishing a vision for a ‘Sound material-cycles society’, at meso/macro-level (Moriguchi 2007). At a global perspective, the literature points out that in most countries, except China, application of CE strategies are concentrated at a single level, most often the meso‐level (Ghisellini et al. 2016).

At both theoretical and practical levels CE stems primarily from the realms of environmental economics and industrial ecology, with a strong emphasis on technological innovation that would enable leaner and cleaner manufacturing technologies as well as better recycling and reuse infrastructure (Ghisellini et al. 2016). Similarly Murray et al. (2017) explain how CE and IE have ‘a shared lineage, with much overlap’ in emphasising economics, while Yuan et al. (2006) claim that the CE originated from the IE closed-loop paradigm. Also, CE is very often discussed through the 3R principles (Liu et al. 2017; Preston 2012; Sakai et al. 2011; Su et al. 2013; Yong 2007).

Consequently, it seems that CE is largely building upon IE’s concepts to establish a new model of economic development, production, distribution, and take-back of products. A way to establish this new paradigm at meso-level can be identified as industrial symbiosis. Chertow and Lombardi (2005) position IS as a subfield of IE that enables companies to think beyond individual operation boundaries and capture cross-organisational synergy opportunities for efficient use of material, energy, and facility resources at a broader systems level. In this way, collaboration between various industrial enterprises can achieve circulation of materials, as waste from one company could eventually become raw material for another (Chen and Ma 2015). Such approaches can also be extended to urban areas, where resource synergies are found between industry, residential, commercial areas, and urban transport (Baas 2011).

CE development in cities, regions, or nations (macro-level) involves the integration and the redesign of four systems: the industrial system; the infrastructure system; logistics services organisation; and the cultural framework and the social system (Mirata and Emtairah 2005; Feng and Yan 2007; Ness 2008). Such “systems integration” approaches, including interdependence and closed loops, have recently been developed (Van Berkel et al. 2009; Jiao and Boons 2014), and have influenced the implementation of eco-towns in several regions around the world, such as Kawasaki city in Japan, and the Hammarby Sjöstad district in Stockholm, Sweden (Van Berkel et al. 2009; Iveroth et al. 2013).

Successful cases of CE implementation, mentioned by Ghisellini et al. (2016), stress the fact that the transition towards CE can be realised only with the involvement of all actors within the society and their capacity to link and create suitable collaboration and exchange patterns. Also, there is a basic requirement for an economic return on investment, in order for the CE paradigm to provide suitable motivation to companies and investors. Obviously, interdependence of all actors is paramount for a CE to work, and the links within a CE system are more than economic and material (waste/resources) but also organisational (Ranta et al. 2017) and environmental (Moriguchi 2007). Song et al. (2013) exemplify that the structure of circular industrial value chains are complex and include a number of interrelated subsystems, such as internal production system, organisational system, technical capabilities system, environmental control and compliance system, energy saving and emission reduction system, supply chain system, etc. Therefore, the characteristics of the whole system are influenced by the individual characteristics of each subsystem, but also by the mutual coupling and interrelations among the subsystems. Moreover, the resulting CE system needs to be effective and efficient to provide the anticipated results of reduced material and energy consumption (and related environmental burdens relief). Whereas efficiency relates to throughput, and can be associated with any of the subsystems and the system as a whole, effectiveness is associated with the holistic functioning of the system. Webster (2013) argues that CE will optimise the system as a whole, versus maximising components or one element of a system. A great enabler in harmonising and fine-tuning such a complex system can be sought in cutting-edge technologies, such as big data and internet of things (IoT), which have the potential to leverage the adoption of CE concepts by individuals, industry, organisations and finally the society as a whole (Nobre and Tavares 2017).

From a sustainability point of view, CE has been found to lack on considerations towards the social dimension (Broto et al. 2012; Jiao and Boons 2014; Murray et al. 2017). However, Murray et al. (2017) identified that some elements of relevance do exist in the CE narrative, referring to the maximisation of ecosystem functioning and human well-being, apart from the obvious tenet of job creation (locally). Moreover, the strong “material” focus in the current narrative of CE seems to preclude wider systemic considerations of sectoral approaches to CE (Haupt et al. 2017). While often the resource optimisation at the manufacturing and end-of-life phase is targeted by CE, as seen by the implementation of CE strategies so far, there is a need to move towards CE also in the agri-food (Marsden and Farioli 2015), built environment (Ness and Xing 2017) and mobility sectors, as these constitute main contributors to environmental impacts (Tukker 2006; EEA 2016a).

## Policy landscape in the European Union

Policies for resource efficiency, incorporating elements of Circular Economy, can be traced back to the 1980s and 1990s in German and Japanese strategic decision-making, largely influenced by the intriguing and then new concept of the closed-loop economy, as presented in the Spaceship Earth analogy of Boulding (1966), and later developed by Stahel and Reday-Mulvey (1981).

**Fig. 2:**
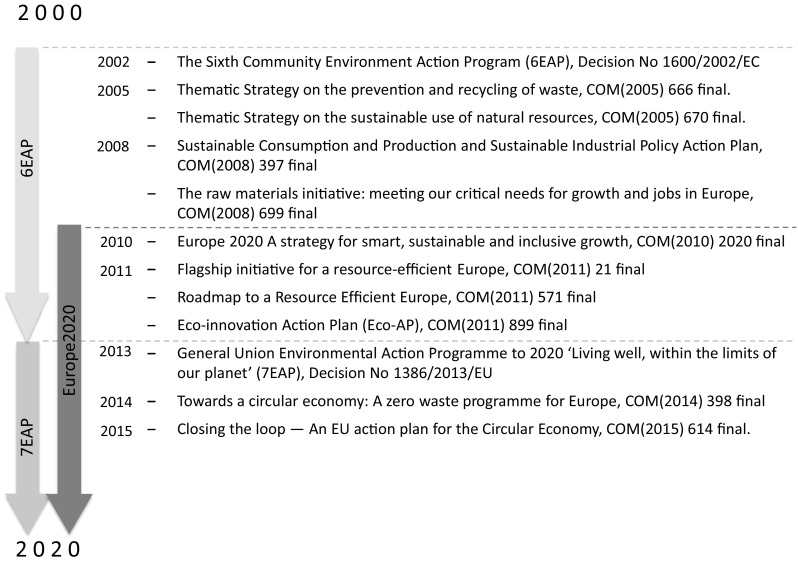
Timeline of EU resource-related strategies

Over the last 15 years the strategic resource policy direction of the European Union gradually turned towards the sustainable use of natural resources, increasing resource efficiency in the economy and scaling up the recycling and prevention of waste, while simultaneously aiming at sustainable levels of economic growth. Figure 2 illustrates a timeline of initiatives taken by the EU in the last 15 years to address resource efficiency.

The latest attempt of the EU to promote a Circular Economy resulted in a package of proposals, including a comprehensive Action Plan (COM (2015) 614 final) and regulation amendments. The aim of the CE package is to improve competitiveness of EU business by shielding industries against potential resource scarcities and volatile prices, and help to create new business opportunities and innovative ways of production and consumption. Circular Economy is expected to create local jobs in the EU at all skill levels in the workforce and opportunities for social integration. It is particularly stressed in the Action Plan that economic actors, such as business and consumers, are the key drivers in the transition process. However, local, regional and national authorities are encouraged to act as catalysts in this transition, with the EU playing a supportive but fundamental role, ensuring that the right regulatory framework is in place for the development of Circular Economy in the single market. The EU CE Action Plan outlines potential policy interventions that would enable the development of CE in the EU (see Fig. 1).

The number and complexity of interactions among actors in a Circular Economy create a complicated policy landscape, which inevitably extent across the different parts of production and consumption systems and affect directly or indirectly several other parts in the value chain. Such interaction networks might as well extend in different geographic locations within or between Member States.

In Table 1, existing mandatory and voluntary legislation relevant to the Circular Economy at EU level is categorised by life cycle stage and further distinguished by having a direct or indirect effect on Circular Economy principles (policies written in black have direct effect on the CE and policies written in grey have indirect or partial effects). This visual representation helps to identify gaps in policies addressing different life cycle stages. Mandatory legislation includes EU regulations and directives which are binding for the Member States. Failing to comply will result in administrative, economic, or even criminal penalties. Voluntary measures, such as eco-labelling and Green Public Procurement (GPP), describe a general regulatory approach but are not binding, and each Member State may decide the level of their application—from full implementation to no implementation at all—without any penalty. Legislation with a direct effect on CE would be one that targets specifically a material for resource efficiency, e.g. waste legislation prescribes waste prevention, preparing for reuse and recycling as the most resource efficient options and sets legally binding targets.

There is a high concentration of mandatory EU legislation towards the end of the life cycle with the aim to limit resource loss and increase the circulation of materials mainly through recycling. Policies targeting consumption are particularly limited and mostly affecting indirectly resource efficiency. There is a plurality of directives and regulations governing production processes at EU level, but the majority does not explicitly target material resource efficiency, and as a result a policy gap is observed at this life cycle stage as well. However, the fact that some policies do exist at that level is considered positive, as material resource efficiency considerations could be easier added in an existing policy than creating an entirely new policy framework from scratch (for instance by improving criteria for public procurement and eco-labelling so that material resource efficiency becomes more prominent).

At Member State level, individual countries have the freedom to devise their own resource efficiency agenda as long as they do not counteract EU regulations. Recently, some Member States decided to take resource efficiency policies a step further, leap-frogging far from the existing EU policies. Table 2 summarises a few ambitious policies at Member State level, aiming at increasing resource efficiency.

**Table 2:** New policy approaches in EU Member States promoting the Circular Economy

Member State	Policy measure	Application
France	Act on consumption and preventing planned product obsolescence The Act (Law no. 2014-344) addresses product durability and aims at preventing planned obsolescence. The law includes articles related to the lifespan of consumer goods, including the introduction of extended product guarantee from 6 months to 2 years; and the obligation of retailers to inform customers about the time horizon that spare parts will remain available for a product in question (EEA 2016b)	Mandatory National
Spain	Reuse targets for Waste Electrical and Electronic Equipment (WEEE) In its new Waste Management Plan 2016-22, Spain sets a target of 50% municipal waste to be prepared for reuse or recycled, followed by a specific target of 2% for preparation for reuse in certain waste streams including textiles, WEEE, furniture and ‘other suitable waste streams’ (Ruiz Saiz-Aja 2016)	Mandatory National
Sweden	Value Added Tax (VAT) reduction in repair services The Swedish government suggested a VAT reduction in repair services for a selected group of products (bicycles and shoes). In addition, the government proposed a tax deduction for repair services performed in relation to home renovations (IVA 2016)	Mandatory National
Sweden	Public procurement of refurbished ICT equipment by Swedish municipalities Two Swedish municipalities (Gällivare and Laholm) apply specific criteria in public procurement, tendering the provision of refurbished ICT equipment for use in municipal services (Avfall Sverige 2015)	Voluntary Local

## Three policy options for advancing to a Circular Economy

Based on the analysis of existing policies in the EU and the interesting examples of national policies targeting resource efficiency with a novel approach (“Policy landscape in the European Union”), three promising policy areas are identified which could radically increase resource efficiency and pave the way for realising a Circular Economy in Europe. Table 1 illustrates a lack of policies that could drive resource efficiency in the production stage and at individual product level. However, the biggest gap is observed at the distribution and use life-cycle stage. No policy implicitly targets resource use in that stage and as such there is no apparent driver for resource efficiency related to consumption and use of products and services, neither at individual consumer level nor by businesses and public sector stakeholders. Finally, a plurality of mandatory and voluntary policies exists at the end-of-life stage, but mostly relating to the sound waste management and increase of recycling. However, the increase of recycling—by mandatory policy targets—cannot guarantee overall resource efficiency, since the type and use of the recyclates is a key defining aspect of CE. Recycling should result in good quality materials that would be able to circulate back to the economy and substitute virgin material resources. Low quality recycling is not able to fulfil the principles of CE, and therefore, is undesirable. In this respect, a gap in policies can be identified also at the end-of-life stage (Table 1) for policies that would promote and upscale quality recycling and market mechanisms that facilitate the reintroduction of recycled materials into production processes.

Having identified the gaps in required policies, in the following “Policies for reuse, repair and remanufacturing, Public procurement for resource efficiency, Strengthening secondary resource markets” the relevance of these policy areas will be discussed and the potential paths of future policies’ application will be analysed. Although the identified policy areas are three, the actual number of policy interventions per area is higher (i.e. different policy instruments within the same policy area). For this reason the discussion in the following sections is developed by policy area and not specific policy instruments.

### Policies for reuse, repair and remanufacturing

In a Circular Economy, the ultimate goal is to retain the inherent value of products by utilising a product for as long as possible and within the shorter loops of material circulation, i.e. reuse, repair and remanufacturing. Therefore, a place to start is by investigating the most appropriate way—in term of policies—to establish increased material circulation in short loops.

#### Durability and reparability policies

Improved design would render products more durable and facilitate reuse, repair, upgrade and remanufacture. Durable products, i.e. products that are designed to last longer or be easily repaired, hold the greatest potential for resource savings by maintaining their operational utility longer while at the same time benefiting their users by saving money on replacement purchases. A formal or legally binding definition of durability does not exist, a fact that makes any policy consideration challenging. However, durability is inextricably linked with the reparability of a product and comprises an integral design feature that makes possible to maintain, upgrade and reuse a product. In this respect, durability and reparability can be considered as the two faces of the same coin and should be addressed together (Maitre-Ekern and Dalhammar 2016).

In addition to product design considerations, two other issues are of high importance for the durability and/or reparability potential of a product: (1) access to spare parts at a reasonable cost; and (2) access to relevant repair information. Despite the importance of these two enabling conditions for reparability, it is observed that manufacturers often try to prevent other actors on the market from having access to spare parts, or from refurbishing and re-selling old products. Moreover, manufacturers hesitate to release product information that could facilitate repair activities, either directly by the product users or third party repairers (i.e. not affiliated with the manufacturers, the so-called “gap-exploiters”; see Whalen et al. 2017). There are also several other ways to limit the reparability of products, such as making spare parts in old products incompatible with similar parts of more recent models of the same product, and preventing disassembly using specific tools and screws or using chemical adhesives to fit the parts together (Maitre-Ekern and Dalhammar 2016).

There is a variety of policy approaches to foster durability and reparability in products, either directly or indirectly. Taking into account that more than 80% of all product-related environmental impacts are determined in the design phase (Tischner et al. 2000), one potential approach is to regulate durability and reparability in a direct manner, such as setting clear mandatory requirements on product lifetime, product reparability and eventually product recyclability (considering also the end-of-life of the product). To date, there is very little experience with standards on reuse and repair (Wilts et al. 2016), but an instrument that has been used successfully in the past for implementing energy efficiency in products (the Ecodesign Directive 2009/125/EC) can be employed to regulate material resource efficiency as well. However, concerns about the effectiveness of such an instrument are raised taking into account the long time period required, from setting technical specifications for durability and reparability until the implementation phase (Wilts et al. 2016). Other potential approaches that could directly influence durability include integrating durability information in energy labelling, setting durability requirements in public procurement criteria, or entering into voluntary agreements with industry over durability issues (Dalhammar 2016).

Innovative policy approaches that could affect durability and reparability indirectly include (1) the adoption of legal measures that make products easier to repair or upgrade, for instance by requiring that spare parts are made available for a number of years after product purchase; or (2) mandating manufacturers to provide information to repairers and remanufacturers that can facilitate repair and remanufacturing practices (Maitre-Ekern and Dalhammar 2016).

An important aspect that requires close attention in policy-making for reparability is the cost of the spare parts. Usually, the major reason for consumers to purchase a new product rather than repairing their old is related to the associated repair costs. The cost of one spare part is often just below the price of the new product, and it is rather tempting for the consumer not repairing the old product but buy a new one instead. The added cost of all spare parts may quickly exceed the price of the product itself (Maitre-Ekern and Dalhammar 2016). Therefore, a careful consideration of the pricing and availability of spared parts should be prioritised and appropriate solutions should be devised in cooperation with manufacturers and retailers.

#### Remanufacturing policies

Apart from extending the life-time of products through durability, maintenance and repair, another promising area of Circular Economy is remanufacturing, which enables the refurbishment and reconditioning of products to a level of quality, functionality and warranty that equals and competes with brand new products, while retaining the maximum of the value and resources in the old products (Matsumoto et al. 2016). Products that have the highest potential for remanufacturing show the following characteristics: (1) stable product technology, (2) stable process technology, and (3) a physical lifetime of critical subparts that is substantially longer than the actual life-time of the product itself (Matsumoto et al. 2016). Taking into account these characteristics, attention should be paid whether the remanufacturing process may contribute to overall resource efficiency, especially in relation to energy use of products throughout their life cycle (Gutowski et al. 2011).

Despite the fact that remanufacturing is increasingly attracting attention worldwide, there is still a wide range of issues that require careful consideration and further research before any policy interventions can be suggested. There is a gap in the knowledge of general framework conditions for products that return to the market after remanufacturing operations. It will not always be clear who puts a remanufactured product on the market, and under which conditions remanufacturing may impede the rights of the original producer. Further unresolved legal issues include compliance to several EU rules, especially those concerning Extended Producer Responsibility (EPR). Another question is whether remanufacturing by third party operators actually breaches intellectual property of OEMs over their products. The former issue is most likely to be considered of high importance by remanufacturers, as they do not want to become ‘producers’ in the meaning of some EU Directives, and thus become economically responsible, for e.g. collection and recycling of waste products, among other responsibilities (Maitre-Ekern and Dalhammar 2016).

**Fig. 3:**
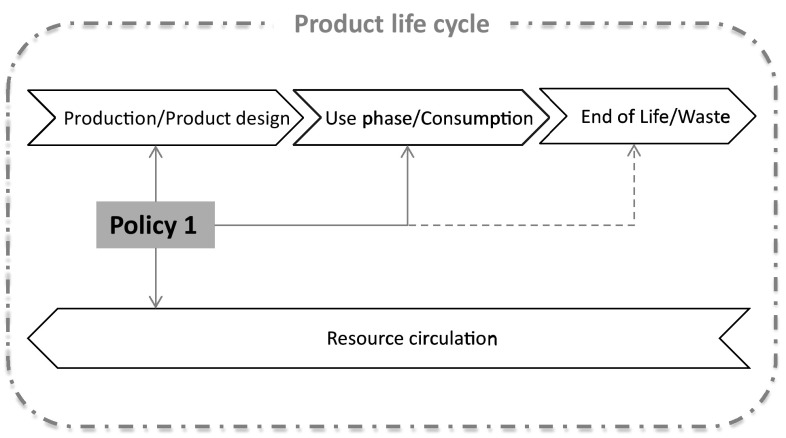
Influence of reuse, repair, and remanufacturing policies on a product life cycle

Concluding, policy instruments within this policy area, as discussed in this section, can have a direct impact on the design of products and the availability of spare parts, while enabling repair and reuse of products that circulate within the economy. Figure 3 illustrates the direct influence of such policies on a product life cycle and indicates that some indirect relation can also be achieved at the end-of-life, for instance by incorporating additional recyclability standards on top of durability and reparability mandatory ecodesign rules. Policy interventions at this stage of a product life cycle can, therefore, exert the highest impact on the resource-saving potential of products and promote resource efficiency.

### Public procurement for resource efficiency

Governmental actors and public authorities, apart from their role in policy decision making, regulation, administration and monitoring, exercise also a significant leverage on the market as a large consumer of goods and services. The significance of public procurement has increased over the last decades, and the EU is emphasising its role as a policy instrument for demand-side innovation (Edler and Georghiou 2007).

The way the demands are made in public contracts affects the outcome of the resource efficiency potential of the purchase and its innovation. Furthermore, the level of uptake by governmental bodies and the specificity of criteria play also an important role. The uptake of resource efficiency-relevant public procurement is highly variable and relates to the size of the governmental entity, the ambition and strategic approach, the lack of knowledge, and the actors involved in the procurement process (Bratt et al. 2013; Guenther et al. 2013; Preuss 2007; Marron 2003). A case study on Norwegian municipalities and regions shows a correlation between the size of the municipality and Green Public Procurement (GPP), where GPP is significantly more established in larger municipalities, while smaller municipalities might have to collaborate with external market actors or other municipalities. Larger municipalities more often have a dedicated purchasing department and a purchasing strategy, which is considered important to develop effective public procurement processes (Michelsen and de Boer 2009). Also Marron’s (2003) findings show that GPP is more effective when the government sector is a large co-ordinated purchaser of products. Furthermore, a reason to why GPP is not used to full potential in municipalities is the obstacles set by different key actors within the public procurement process (Günther and Scheibe 2006; Brammer and Walker 2011). Market actors, national agencies, citizen organisations, procurement department, finance department, environmental department and users are actors often involved in the process and can all be sources for ‘disturbing factors’, such as no clearly defined goals, no explicit regulations, lack of information, and large knowledge gaps (Günther and Scheibe 2006).

The possibility to use GPP as a pro-active tool for resource efficiency is not fully utilised. This is mainly due to short-term decisions, often hastily made, and GPP criteria that are often adopted having in mind only existing products and services on the market, while innovative Product-Service System (PSS) solutions are not stimulated. To overcome this barrier and allow innovative products or services to enter the market, governments have a big role to play by promoting a different approach to procurement, the so-called public procurement for innovation. Suppliers with the potential to provide innovation indicate that the lack of interaction and understanding with the procuring organisations and over-specified demands in tenders consist the main barriers for innovation procurement (Uyarra et al. 2014).

Finally, Witjes and Lozano (2016) studied the possibility of introducing a new form of procurement combining the aspects of PSS with the functional demands of public organisations. In this case, the main objective of the tendering negotiation between supplier and procurer switches from product oriented procurement to PSS (for PSS definition see Mont 2002), thus switching from a price per product unit to price per delivered service, as the functional unit of the tender negotiations (Witjes and Lozano 2016).

**Fig. 4:**
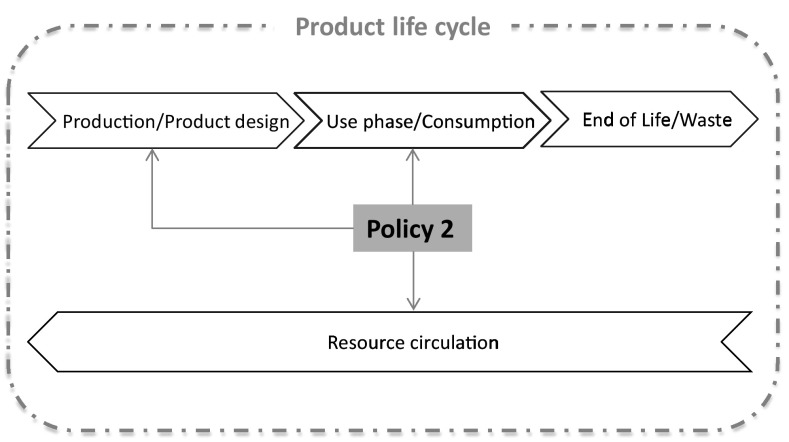
Influence of public procurement for resource efficiency on a product life cycle

Concluding, GPP and public procurement for innovation can have a direct impact on the consumption of products and services and directly influence the design and configurations (product/service proposition and delivery methods) that should comply with the procurer’s criteria. Furthermore, resource efficiency demands that might include repaired and/or reused equipment would have a direct effect on material and product circulation in the economy as presented in Fig. 4. Therefore, it is important to promote the creation of relevant resource efficiency criteria (even beyond the traditional notions of recycled content in new products) and integrate them into mandatory GPP requirements, at local, regional or national level.

### Strengthening secondary resource markets

Despite the fact that it is relatively commonplace to extract additional value from end-of-life goods through recycling or recovery operations, this is generally not reflected in the mechanisms of product design, pricing and market regulation (Stahel 2016). Markets for secondary raw materials are recognised by the European Commission as a critical area for action and improvement (European Commission 2015a). As the markets for recyclables are expanding nationally and internationally, several issues arise concerning the functioning and properties of such markets and their actual contribution to the Circular Economy vision (Kama 2015). Nicolli et al. (2012) point out that there is a significant risk of high search and transaction costs associated with recyclable materials in secondary markets, related to incomplete information. There is usually a lack of information concerning the quality and properties of potentially recyclable or reusable materials and products. In addition to this, the provided information is usually asymmetric, in the sense that the supplier holds a negotiating advantage by knowing more about the quality or properties of the material or product than the potential buyer. In such cases, a broad range of policy instruments can be used to support the markets. The establishment of harmonised quality standards for recycled materials and/or certification schemes could be useful in overcoming such barriers (Finnveden et al. 2013).

Apart from the need to communicate the quality of the recycled materials on the market and improve the functioning of such markets, another critical aspect concerns the necessary initiatives required for achieving high quality recycling. Although advanced recycling technology is vital, equally important are the operations preceding the recycling plant. The design and use of certain materials in a product, the collection systems (level of separate and clean collection of materials), and the efficiency of sorting operations (sorting out single materials from mixed waste fractions), are considered fundamental in increasing recycling in quantity, quality and efficiency (Gregson et al. 2015).

Furthermore, some existing technologies of waste treatment do not align with the Circular Economy goal of recovering secondary resources from waste (Gregson et al. 2015). Policy incentives should facilitate a shift away from incineration and low quality recycling towards developing cleaner material cycles and high quality recycling. However, such incentives should be carefully considered not only to provide direct support for recycling that reduces the downstream impacts of technological externalities (i.e. cost-effective recycling), but also influence the upstream conditions to internalise such externalities (i.e. design for recyclability) (Söderholm and Tilton 2012). For instance, if resources are allocated towards the development of sorting technologies that enable the recycling of composite waste fractions, product designers and manufactures will be discouraged from redesigning their products, as the advanced sorting technologies will take care of their composite products.

A related concept that would incentivise a more pro-active attitude from industry is that of Extended Producer Responsibility (EPR) (Lindhqvist 2000). Currently, the producer responsibility is organised at sectoral level and the costs for the collection and recycling are shared among the participating companies, based on the amount of products put on the market. This approach results in overall reduced costs but it lowers the ambition of individual companies to develop more circular products, as a company would have to bear the costs of improved design and production changes while the benefits of the reduced end-of-life costs would be shared with all other companies in the market. If producers within an EPR system need to take care of discarded products irrespective of brand, there is practically no incentive to invest extra resources on improving their products’ design to reduce the impacts from end-of-life management (Van Rossem et al. 2006). As a result, producers increasingly believe that EPR rules can no longer provide design incentives for recyclability (Dalhammar 2016).

The way the current EPR systems are set up involve several actors in the take-back and recycling systems, and the valuable waste materials will likely end up to third parties instead of the producers themselves. In addition, actors outside the EPR systems are striving to appropriate more valuable waste materials (Kunz et al. 2014). So, producers cannot reap the benefits of improved ecodesign themselves, as it is most likely that they will not get materials back. Therefore, if the EPR system is set up by a company/producer as an individual system (Individual Producer Responsibility) then the producing companies might find it beneficial to reduce the costs of recycling by better product design.

Concerning the issue of inducing design changes, importing companies may participate in take-back and recycling schemes fulfilling their EPR requirements, but apart from managing the cost-effective recycling of the products, they have absolutely no power to influence the design in the place of origin, probably far away from the point source of waste. Therefore, a legal requirement for design for recycling may be more effective, than the participation to an EPR system (Finnveden et al. 2013).

**Fig. 5:**
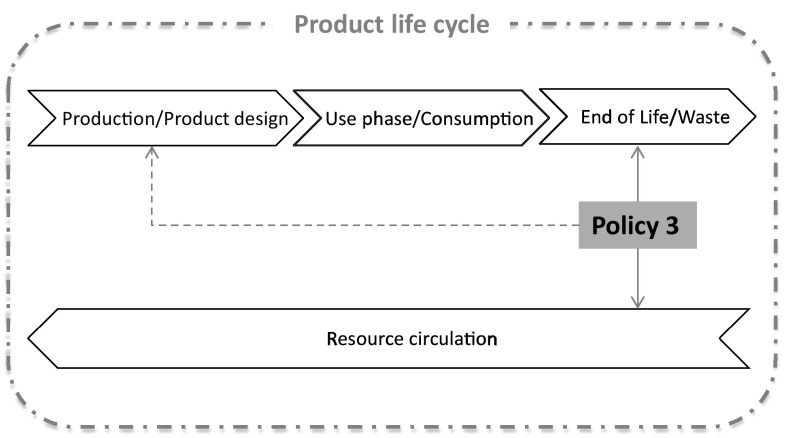
Influence of waste market-related and EPR policies on a product life cycle

Concluding, the policy instruments discussed in this section can have a direct impact at the end-of-life stage of products influencing strongly the reintroduction of valuable materials and parts back in the economy. Figure 5 illustrates the direct influence of such policies on a product life cycle and indicates that some indirect relation can also be achieved at the production stage, for instance by influencing the design of products for recyclability together with related standards and certifications at an early stage of production (even within the material selection process). Policies within this area include: (1) a variety of instruments for increasing information of material content and material quality in products and/or secondary raw materials, such as standards, certifications and product passports; (2) instruments for facilitating the market of secondary materials and the transboundary shipment of waste for increased recycling, taking advantage of economies of scale (studies have already been completed on behalf of the European Commission addressing such issues, e.g. see European Commission 2016); (3) policies promoting a harmonised and inclusive EPR that incentivises producers to apply ecodesign innovations and share the economic burdens of participation according to real resource efficiency improvements; and (4) policies supporting the development of appropriate waste infrastructure across the value chain (from collection to recycling output) in order facilitate clean and homogenous material fractions.

## Policy mix for an effective circular approach

**Fig. 6:**
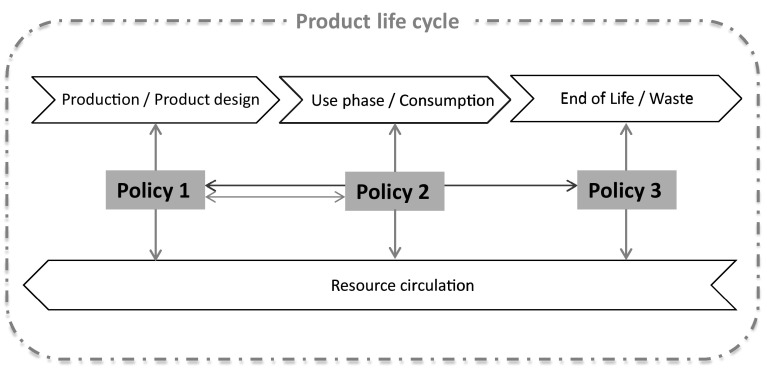
Influence of the three individual policy areas on a product life cycle

In the previous sections, three policy areas with high resource efficiency potential were identified as lacking specific attention in the current policy landscape of the EU, and a variety of interventions within these policy areas were discussed. The policies can individually induce certain desired outcomes and increase the potential of resource circulation across the different stages of a product’s life cycle, depending where the focus of each policy is directed. However, it is apparent that each of these policies can have synergistic effects if applied in conjunction with another policy at a different life cycle stage. For example, although EPR rules strongly influence the collection and recycling of products, they fail to induce the necessary product design changes (Huisman 2013; Richter and Koppejan 2016) that would increase the volume, quality and efficiency of recycling, and ultimately lead to increased material circulation in the economy. For this reason, a combination of mandatory ecodesign rules within an improved EPR system seems like a more effective policy intervention. Figure 6 outlines the plurality of relations among the policy instruments discussed so far, both between the policies and across the product life cycle.

Identifying potential synergies between policy instruments, targeting a wider socio-economic goal such as the transition to a Circular Economy, generates inevitably the question: What is the most effective way to combine these instruments and what the potential outcomes could be? Applying one policy instrument would most likely change an individual driver, but would risk prompting unintended outcomes that change other drivers, and ultimately these changes would counteract or even neutralise the intended effect of the policy instrument. For this reason, a more complex approach needs to be taken in the policy making field, taking into consideration a systemic approach by developing a mix of policies that is targeting a specific outcome.

In a systems perspective, policies ideally would be designed to reinforce positive feedback loops that would escalate resource efficiency in the life cycle stages of production/consumption subsystems, until reaching the desirable goal (initial target setting) of CE. Subsequently, the system would need to readjust in a reconfigured state by appropriate policies introducing balancing feedback loops (Meadows and Wright 2008). In this respect there is a need for a predictive/expecting policy making process, which is not only responding to the state of the problems without acknowledging the drivers and impacts of the policies upstream and downstream the implementation process.

However, designing, implementing, and evaluating a policy mix is much more difficult than individual policy instruments. Actual political processes, affecting the dynamics and path dependencies of legislative periods, pose a barrier in strategic and long-term implementation procedures of policy mixes (Howlett and Rayner 2007).

To successfully respond to, and be adapted to the specific context of a policy vision, the development of policy mixes needs to consider a whole range of related issues (Del Rio and Howlett 2013; Howlett and Rayner 2007): (1) the full range of available policy instruments, as outlined in “Three policy options for advancing to a Circular Economy”; (2) the full cost of policies, including implementation costs, transaction costs and compliance costs; (3) avoid negative interactions between single policies (i.e. instruments already in place and new ones), but emphasise mutual benefits with existing policies; (4) combine instruments to mitigate side-effects; and (5) pay close attention to the political processes during the design and implementation of the mix.

Therefore, a comprehensive policy mix needs to go beyond just combining loosely related or unconnected individual policy instruments to be successful in practice. For increasing its implementation feasibility, it is necessary to consider both the consistency and coherence of the instruments linked in the policy mix. While consistency refers to the absence of conflicts and contradictions, coherence refers to ensuring synergistic effects and positive interactions between instruments as well as between different policy and administrative levels (Rogge and Reichardt 2013).

According to Meadows and Wright (2008), there is a set of well-recognised principles and rules that can be applied in interventions at complex systems. First and foremost, there is an absolute requirement for a clear vision for the future change in the system. Apart from the vision, a transition pathway and a novel paradigm (alter state) are considered essential. Appropriately adapted rules and restrictions are needed to set the framework of the novel system, while a level of self-organisation should be allowed for new actors to enter the system, which will complement the subparts that do not exist today in a linear economy or they are underrepresented (e.g. repairers, scale remanufacture, take back systems public or private, or even individual at company level, etc.). It is imperative to strengthen information flows that will enable ardent communication between the nodes of the system (and subsystems), and reinforce feedback loops. Lastly, it is important to account for delays in the system’s response, as changes happening at a certain timeframe not necessarily accrue results in a predicted fashion, so that a responsive action can be taken timely at another node in the system.

Givoni et al. (2013) present a heuristic ex-ante framework for developing policy mixes, which comprises three basic principles: (1) objectives and targets; (2) causal theory and measure inventory; (3) dynamic ex-ante appraisal and packaging. To reduce the risk of burden-shifting from one life-cycle stage to another, a wide systems perspective is required in the process of designing a policy mix (Ekvall et al. 2016). Consequently, as the implementation of a certain policy mix proceeds, ex-post monitoring can reveal the effectiveness and efficiency of the mix and how (or if) it can be further readjusted to fit its intention (Givoni et al. 2013).

**Fig. 7:**
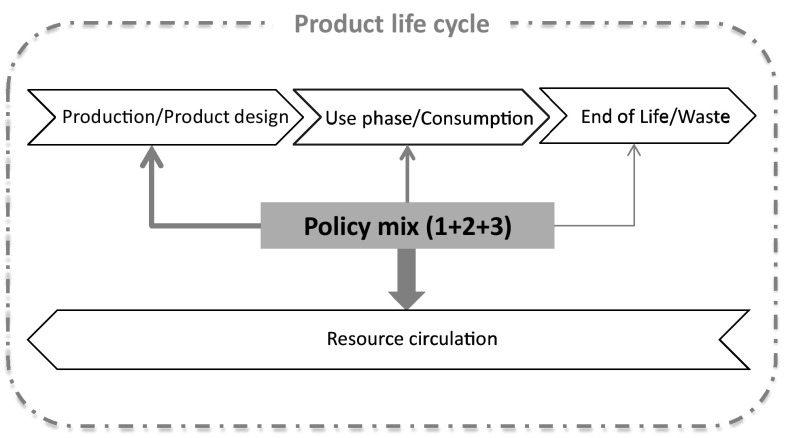
Influence of the policy mix of the three policy areas on a product life cycle

Taking all the above into account, a carefully designed policy mix would result in a strengthened policy framework with a significant influence towards saving resources and increasing the volume and quality of circulated materials and products in the economy, realising the vision of CE. The direct effects of the policy mix would be expected to have higher impact than what a mere combination of different policy instruments would be able to achieve, and would target proportionately all life cycle stages. According to the core principles of CE, the shorter loops should be a priority and thus ideally favoured by the synergistic effect of the policy mix. A schematic vision of a policy mix approach is presented in Fig. 7.

A possible example, among a great variety of desired policy scenarios, would be: mandatory ecodesign rules for reparability together with material and parts certifications would enable increased reuse of the product AA. Carefully designed GPP criteria would favour the uptake of the reparable and reusable product AA, while novel EPR rules for the collection and refurbishment (instead or recycling) of product AA would retain additional value of the product and maintain the resources embedded in the product intact (or with minor modifications). The participation costs to this EPR system will reward companies that are able to repair and reuse their products, minimising any rebound effects, and burden companies that only recover the easy parts and let the remaining product value go to waste.

## Conclusions

Resource efficiency strategies have gained increased attention in recent years from policy makers and businesses alike. The idea of CE, where resources are retained in products and their utility is extended as much as possible, while materials are preserved and re-circulated back to the economy, constitutes the new approach in policy and business circles to tackle the increasing pressures on resource availability and prices. The EU recently introduced the Circular Economy Action Plan (COM(2015) 614 final) outlining future policy directions for resource efficiency adopting a holistic approach, combining a variety of future policy considerations across the life cycle of products.

The current EU policy landscape is rather waste-centric with a plurality of waste-related directives and regulations, focusing on the promotion of a ‘waste hierarchy’ management system. Although, ideally, such a system would lead to waste minimisation and increased reuse and recycling, in reality the current situation in EU is far from this. The majority of product-related policies fail to incorporate any material resource efficiency clauses in a meaningful way. Moreover, a striking gap in consumption-related policies explains the persistent linearity of the current economy, where products become almost deterministically waste and all other resource and value conservation options (e.g. repair, reuse, etc.) remain marginal.

Against this background, three policy areas are identified in this article showing a significant potential for promoting higher resource efficiency throughout the life cycle of a product. These include (1) policies for reuse, repair and remanufacturing; (2) GPP and procurement for innovation; and (3) policies for facilitating the efficient functioning of waste markets and promoting EPR. All relevant policy measures within these policy areas have the potential to influence in a direct way the resource efficiency of products and services, reflecting the core principles of CE and reaffirming the goals of the resource efficiency agenda in the EU.

The shift towards CE would be enabled by the development of policy mixes, as highlighted in “Policy mix for an effective circular approach”, rather than by singular policy instruments put side by side. Policy mixes are generally better equipped to tackle the complexity of systemic challenges, such as the transition to a “new” socio‐economic system. Although a large arsenal of potential policy measures exists today, their implications and unintended side-effects have not been accounted for satisfactorily in research and further investigation is required, firstly at company level (or sectoral level), case by case, and ultimately across the whole economy. Individual policies as well as policy mixes need to be rigorously evaluated for potential rebound effects which could undermine the transition trajectory to a Circular Economy.

Furthermore, policy interventions are required in different levels, from local and regional to national and international, to tackle the challenges of CE in the most effective way. For example, innovators that already embrace CE principles in their business models would need sufficient assistance from the policy environment they operate, to scale up and be able to compete at a national/international context. This “bottom‐up” approach is expected to highlight important issues for future policy research.

Research gaps are identified in policy interventions targeting the inner circles of the Circular Economy, i.e. the shortest loops that retain most of the value in products, namely reuse, repair and remanufacturing. More specifically, research is needed on the definition and implications of repair and durability standards; ecodesign requirements for durability and reparability and how these can be developed, as well as on consumer preferences with regards to resource‐efficient products. Also, the availability of spare parts and their cost, unhindered movement of products for repair as opposed to waste shipments and intellectual property considerations of remanufactured products require deeper understanding. Finally, the role of producers and third party operators in relation to responsibility for second‐hand products on the market, and establishing effective take-back systems for repair and remanufacturing deserve further attention.

“Traditional” approaches to resource efficiency, i.e. recycling, require further research in terms of establishing well-functioning waste markets and identifying conditions for doing so; developing standards and improving the traceability of secondary materials (e.g. for chemicals contents and composite materials); identifying optimal and economic use of waste, and conditions for appropriate alternative waste management options—reuse vs. recycling vs. energy recovery; etc. Better policies are also needed for rationalising and streamlining rules for transboundary shipments of waste, and for clarification of waste‐related definitions.

Finally, in relation to the overall sustainability benefits of a fast transition to the Circular Economy, literature reveals that despite CE’s central role in increasing the sustainability of systems, there are still issues to be addressed more thoroughly in the future. Geissdoerfer et al. (2017) concluded that in most cases CE has a synergistic or conditional effect on sustainability, meaning that CE can be viewed as a precondition for increased sustainability, while sometimes there can be trade-offs in the approach of CE, having costs and benefits in regard to sustainability (Andersen 2007). Although CE is primarily viewed as a sustainability solution for industrialised countries to reduce their material and energy intensity, this does not necessarily mean that the same solutions would have the same outcomes in other parts of the world. Especially in relation to the unequal distribution of production and consumption locales in the global market, where intense resource and labour exploitation usually does not coincide with the major consumption centres. Ultimately, there is need for further research to stress the social dimensions of CE (as for instance in Moreau et al. 2017) and to develop improved metrics of environmental and economic costs and benefits under the light of increasing overall sustainability at global scale.

## Footnotes

1 The database of the Official Journals of the European Union.
